# Immune Biomarkers for Checkpoint Blockade in Solid Tumors: Transitioning from Tissue to Peripheral Blood Monitoring and Future Integrated Strategies

**DOI:** 10.3390/cancers17162639

**Published:** 2025-08-13

**Authors:** Ioannis P. Trontzas, Konstantinos N. Syrigos

**Affiliations:** Third Department of Internal Medicine, Sotiria Hospital of Respiratory Diseases, National and Kapodistrian University of Athens, 152 Mesogion Ave, 11527 Athens, Greece

**Keywords:** immune biomarkers, cancer immunotherapy, peripheral blood, tumor microenvironment, immune checkpoint inhibitors, integrated biomarker profiling

## Abstract

Immunotherapy with immune checkpoint inhibitors has changed the treatment landscape in many solid tumors. Despite the unprecedent success, many patients will develop primary or secondary resistance to treatment or will hold up therapy due to the emerging immune-related toxicity. Traditionally, tissue-based immune biomarkers, such as PD-L1 expression, have been used to select patients who will benefit most from immunotherapy. However, these markers demonstrate major limitations, such as tumor heterogeneity and sample constraints. In addition, they do not reflect the dynamic interplay of tumor and hosts immune response during treatment. Peripheral blood immunomarkers offer a minimally invasive, real-time assessment of the immune system and its interaction with the tumor. Integration of traditional tissue-based and peripheral blood markers coupled with the recent developments in computational platforms, artificial intelligence, and machine learning models may provide more successful biomarkers for prognosis, prediction of immunotherapy-related outcomes, the early evaluation of forthcoming disease progression, and the prediction of the emerging immune-related adverse events. Despite the promising developments in the field of immune biomarkers, several issues including assay standardization, clinical validation, and biological variability should be addressed to improve personalized immunotherapy approaches. In this comprehensive review we provide an update on immune biomarker evolution, and we discuss the current limitations and future directions.

## Simple Summary

Immunotherapy has changed the treatment landscape in many solid tumors with significant improvements in life expectancy of cancer patients. However, many patients do not respond to immunotherapy or will present with resistance to treatment after initial favorable responses, which eventually allow only a small subset of patients to benefit from immunotherapy in the long-term. As a result, better biomarkers to predict which patients will benefit the most, to predict resistance to treatment timely, and to limit forthcoming immune-related toxicity are needed. Traditionally, tissue-based markers, such as PD-L1 and TILs, were used to assess prognosis and response to treatment. However, these markers present inherent limitations including the constraints of biopsy sampling, the heterogeneity of expression of these markers within tumor, and the lack of dynamic assessment during treatment. Peripheral blood markers may be an adjunct tool to the traditional tissue-based markers, bypassing those limitations and offering a real-time monitoring of immune system and cancer interplay during treatment. Despite the promising results of tissue- and peripheral-based immune markers, major methodological limitations, such as assay consistency and method assessments in different labs, hamper their implementation in clinical routine. Recent developments in artificial intelligence and machine learning tools may provide the infrastructure to couple multiparameter approaches and to implement the clinical validation of these markers. Overcoming the obstacles for accurate biomarker analysis may yield major developments in precision oncology in the era of immunotherapy.

## 1. Introduction

Immunotherapy with immune checkpoint inhibitors (ICIs) has transformed the treatment landscape in many solid tumors. Despite the great improvements in clinical outcomes, a significant proportion of patients do not respond to treatment or will develop resistance. Moreover, the emerging immune-related toxicity limits the long-term outcomes of the treatment, especially for patients who will present with severe immune-related adverse events (irAEs). Thus, there is a growing need for the implementation of robust biomarkers to predict prognosis, guide patient selection, and monitor therapeutic efficacy and toxicity.

Recent developments have rendered genomic profiling a cornerstone of precision oncology. Progress in gene biomarkers and signatures has led to the identification of actionable mutations, which guide targeted therapies across various cancer types, and to the recognition of resistance patterns, and, coupled with other approaches, it has unraveled several pathways of tumorigenesis and drug–tumor interactions [1]. Although genomic profiling has significantly advanced our understanding of tumor biology and treatment selection, immune biomarkers give essential insights into the dynamic tumor–host interactions that influence immunotherapy response and, in many cases, it cannot be captured by genomics alone.

Historically, tissue-based immune biomarkers have been the cornerstone for the selection of patients treated with ICIs. Programmed death-ligand 1 (PD-L1) expression, initially introduced for guiding immunotherapy in non-small-cell lung cancer (NSCLC), remains the most used companion diagnostic across several malignancies [2]. Other tissue-based markers, such as the density of tumor-infiltrating lymphocytes (TILs), have shown promising results in the assessment of the tumor immune microenvironment (TIME), with growing evidence supporting their prognostic and emerging predictive value in various cancers [3,4]. Besides PD-L1 and TILs, several tissue-based biomarkers are increasingly recognized for their role in tumor immunity and their influence on immunotherapy responses [5].

Tissue biomarkers provide critical information for the TIME and ICI action. However, their clinical utility is often limited by several factors. One major limitation is that the assessment of these markers requires tissue sampling, which is an invasive approach and often reflects only a small fragment of the tumor heterogeneity. The tumor microenvironment not only demonstrates varying spatial distribution but is also dynamically reshaped during the treatment course [6]. Thus, it is crucial to use biomarkers that reflect real-time changes in the immune status.

Peripheral blood immune biomarkers have emerged as complementary diagnostic tools, offering minimally invasive, active assessment of the tumor–immune system interactions. The variety of blood immune parameters may collectively provide a real-time map into the immune landscape and into the evolving interactions during treatment [7].

Although evidence on the use of peripheral immune biomarkers is growing, their clinical implementation is hampered due to several limitations, such as the cellular heterogeneity, the variability across time points, and the lack of standardized assays and prospective validation studies [7,8]. Furthermore, single biomarkers often fail to fully explain the complexity of the immune response. Thus, a shift towards integrated, multimodal biomarker strategies seems to be crucial. Development of computational tools, large multiplex platforms, machine learning (ML), and artificial intelligence (AI) render integrative approaches feasible; therefore, the coupling of tissue-based and peripheral immune biomarkers to accurately map immune system and tumor interactions seems to be more relevant today than ever before [7,8].

Herein, we aimed to provide an updated overview on the established and investigatory immune biomarkers, their current status, and problems for their implementation in clinical practice. Moreover, we aimed to emphasize the necessity for the adaptation of integrated biomarker approaches, especially in the era of new technological platforms and computational AI tools.

## 2. Tissue-Based Immune Biomarkers

**Figure 1:**
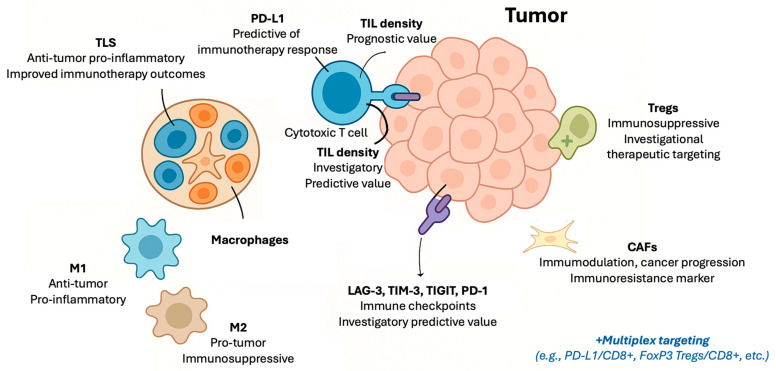
Schematic illustration of tissue-based immune biomarkers in the era of immunotherapy. **Abbreviations:** CAFs: cancer-associated fibroblasts; CD: cluster of differentiation; LAG-3: lymphocyte activation gene-3; M1, M2: macrophage subset; PD-1: programmed death receptor-1; PD-L1: programmed-death ligand 1; TIGIT: T-cell immunoreceptor with Ig and ITIM domains; TILs: tumor-infiltrating lymphocytes; TIM-3: T-cell immunoglobulin and mucin-domain containing-3; TLS: tertiary lymphoid structures; Tregs: regulatory T-cells.

Several tissue immune markers have been studied and some of those have been adopted in clinical practice during the era of immunotherapy, offering prognostic information and guiding treatment decisions (Figure 1). From the early recognition of TILs as a surrogate of immune interactions in breast cancer (BC) and melanoma to the widespread use of PD-L1, these markers have shaped the landscape of decision making in Oncology and some of those have been incorporated as companion diagnostics by the regulatory authorities. Better understanding of the spatial organization of the TIME and of the involved immune system counterparts in recent years highlights not only the progress that has been made but also the challenges ahead. A summary of the currently validated and investigational tissue-based immune markers is provided in Table 1, and a more detailed overview of each marker follows.

### 2.1. Programmed Death-Ligand 1 (PD-L1) Expression

Programmed death-ligand 1 (PD-L1) expression is the most commonly used biomarker to guide immunotherapy decisions in solid tumors. Initially developed to identify NSCLC patients most likely to benefit from anti-PD-1/PD-L1 therapies [9], it is nowadays used as a companion diagnostic for several other cancers, including urothelial carcinoma [10], head and neck squamous cell carcinoma (HNSCC) [11], triple-negative breast cancer (TNBC) [12], gastric and gastroesophageal junction cancers [13], and cervical cancer [14]. In clinical practice it is commonly assessed with conventional immunohistochemistry (IHC) or using automated stainer devices, and its expression is generally associated with better immunotherapy outcomes [2]. On the other hand, the prognostic significance of PD-L1 remains controversial. Despite some evidence suggesting an association with worse survival outcomes in patients not receiving immunotherapy, other studies are inconclusive on this relationship, limiting its utility as a universal prognostic marker [15].

Despite its widespread use, PD-L1 as a biomarker demonstrates significant limitations. It is now well established that its expression is largely heterogeneous, varying both spatially within the tumor and between primary lesions, metastatic sites, and infiltrated lymph nodes, which raises concerns about sampling bias [6]. Moreover, there is not a universal assay platform for all tumors, nor a consistent scoring system, with some drug indications requiring expression per tumor proportion score and others per combined positive score, while the different antibody clones used per assay add further complexity to its interpretation [16]. In addition, PD-L1 expression is a dynamic feature that can evolve under pressure from therapy, thus isolated biopsies at cancer diagnosis do not reflect the dynamic interactions in the TIME [17]. These factors collectively underline the need for complementary or alternative biomarkers that can offer a broader, more dynamic view of the tumor–immune interaction.

The progress made on digital pathology tools over recent years may address some of these obstacles by offering automated PD-L1 scoring and spatial distribution analysis. Early studies suggest that digital image analysis can improve reproducibility and reduce inter-observer variability compared with manual assessment [18]. In addition, the integration of spatial data can provide a deeper understanding into the differential expression of PD-L1 by tumor and immune cells and examine the relationship of PD-L1 in association with other immune parameters and with the tumor architecture. Despite the promising early evidence, clinical adoption remains limited at this stage [19,20].

### 2.2. Tumor-Infiltrating Lymphocytes (TILs)

Tumor-infiltrating lymphocytes (TILs) were among the first immune markers recognized to play an important role in the TIME [3]. The presence of high TIL density has been associated with an active immune microenvironment, which suggests a strong prognostic and potential predictive value for immunotherapy [21]. TILs have demonstrated robust prognostic value across multiple solid tumors, often independent of treatment modality. Most evidence emerges from melanoma studies and from early BC, especially TNBC and HER2+ subsets, where TIL evaluation is recommended by expert consensus as a prognostic marker, though it is not yet used to guide immunotherapy [3,4]. In NSCLC, increased TIL density has similarly been linked to favorable prognosis [22].

There is also emerging evidence regarding TILs predictive potential for immunotherapy. Higher TIL densities have been associated with improved outcomes to ICIs, reflecting a ‘hot’ tumor environment in several studies [21,23].

Despite their promising potential, TILs are not routinely used in the clinic. Besides the lack of standardized scoring, there is a great variability between different observers and laboratories. There is, though, an ongoing effort with consensus recommendations to guide standardized assessment of TILs across tumor types [24,25]. This international pathologist collaborative effort, based on the infrastructure of TIL measurement approaches provided from phase III neoadjuvant BC trials [25], emphasizes the generalizability of steps for TIL counting, such as the selection of proper representative tumor area, the accurate definition of stromal and intra-tumoral areas, the careful microscopy assessment under low-magnification, the determination of proper type of inflammatory infiltrates (e.g., exclusion of granulocytes in necrotic areas), and the classification of TILs in crude percentage groups associated with prognosis or response prediction, while acknowledging the differences arising per tumor type and the special considerations needed (e.g., exclusion of TILs found on alveolar macrophages in NSCLC) [24]. Additionally, there are no consistent assessment methods, varying from basic IHC techniques to more sophisticated multiplex digital assays, rendering comparisons difficult [25]. Furthermore, the varying distribution of lymphocytes within tumors means that small biopsy samples may not accurately represent tumor environment [3]. Emerging digital pathology tools offer promising solutions by enabling automated, quantitative, and spatially resolved assessment of TILs, which may help improve reproducibility [3]. Further studies, testing TILs in prospective settings are necessary to establish more solid conclusions.

### 2.3. Other Tissue-Based Immune Markers

Beyond PD-L1 and TILs, several other components of the TIME have emerged as promising tissue immune biomarkers.

Macrophages are one of the most thoroughly studied components of the TIME. In particular, it is suggested that the balance between specific macrophage subsets, such as pro-inflammatory M1 and the immunosuppressive M2 phenotypes, may influence tumor progression and patient prognosis in several solid tumors. More specifically, it has been shown that high infiltration of M2 macrophages often correlates with worse outcomes, whereas M1 macrophages are generally associated with better anti-tumor activity [26]. Moreover, there are emerging data suggesting that specific tumor-associated macrophage (TAM) subpopulations within the TIME may influence immunotherapy outcomes; however, robust prospective validation remains necessary [27].

Another component of the TIME, the cancer-associated fibroblasts (CAFs), are considered key players in the modulation of immune responses. Generally appraised as promoters of immunosuppression, certain CAF subsets have been linked to poor prognosis and resistance to immunotherapy, especially in studies on NSCLC and skin cancer [28]. However, their use as biomarkers is still under investigation.

Regulatory T-cells (Tregs), a unique TILs subset, are considered critical components on the development of immune tolerance within the TIME. Elevated infiltration of Tregs in various cancers has been associated with poor prognosis, as they hamper effective anti-tumor immune responses and promote immune evasion [29]. Several studies have further characterized their immunosuppressive and tumor-promoting features with Tregs predominant phenotypes of tumor-infiltrating cells being associated with more aggressive disease behavior (e.g., higher proportions of Treg-predominant phenotypes of immune-infiltrates in TNBC and higher grade lesions), highlighting their role in tumor’s growing and immunosuppressive pathways [30]. Similarly, Tregs also influence the response to immunotherapy as their presence can contribute to resistance against ICIs by dampening T-cell activity [31]. Despite not being validated predictive biomarkers, their distinctive immunoregulatory role makes them attractive targets for the development of inhibitors which, coupled with ICIs, may boost immunotherapy efficacy [32].

Tertiary lymphoid structures (TLSs) are considered an emerging tissue biomarker with diverse role on immunomodulation. These structures are ectopic lymphoid aggregates that form within or adjacent to tumors, resembling secondary lymphoid organs. They consist of organized clusters of B-cells, T-cells, dendritic cells, and other immune components, and act on local antigen presentation and to stimulation of adaptive immune responses. The presence of TLS in various cancers has been associated with improved patient survival and enhanced response to immunotherapy [33]. Despite growing evidence supporting their prognostic and predictive value, standardized methods for TLS detection and quantification are still under development, and their incorporation into clinical practice remains limited. Recent clinical studies, such as the PEMBROSARC trial investigating pembrolizumab in soft-tissue sarcoma, are prospectively evaluating TLS as biomarkers for immunotherapy response, representing a significant step toward validating TLS clinical utility in patient selection and treatment planning [34].

Lastly, there are other immune checkpoint proteins, besides PD-L1, that have gained interest as tissue biomarkers due to their roles in tumor immune evasion. Molecules such as LAG-3 (Lymphocyte Activation Gene-3), TIM-3 (T-cell Immunoglobulin and Mucin-domain containing-3), and TIGIT (T-cell immunoreceptor with Ig and ITIM domains) are variably expressed on different components within the TIME and have been associated with T-cell exhaustion [35,36,37]. There are studies suggesting a prognostic role of these proteins and potential utility on predicting immunotherapy outcomes [35,36,37]. However, these markers are currently considered investigational and are not used in daily clinical practice. There are, though, ongoing clinical trials evaluating novel checkpoint inhibitors targeting these molecules which may soon clarify their utility as predictive biomarkers.

Further, continued research is needed to establish the role of these TIME-based biomarkers alongside commonly used markers like PD-L1 and TILs.

### 2.4. Combination Approaches to Tissue-Based Immune Biomarkers

Despite the variety of tissue-based markers, analysis of single biomarkers often fails to fully interpret the complex interactions of immune system and tumor. Thus, approaches exploiting the concurrent use of multiple biomarkers for the improvement of their prognostic and predictive accuracy have gained interest.

The combination of PD-L1 expression and CD8+ T-cells has been thoroughly studied and has demonstrated strong predictive accuracy for immunotherapy in many studies. This combined approach provides an insight into the adaptive immune resistance mechanisms, as tumors are considered to upregulate PD-L1 expression and evade immune attack in response to recruitment of infiltrating cytotoxic T-cells [38]. Clinical studies in various cancers, such as NSCLC and melanoma, have shown that patients whose tumors are characterized by both high PD-L1 expression and high CD8+ density tend to experience better responses to ICIs [38,39,40].

Beyond this, there are several other studies on combination approaches which aim to integrate markers of immune regulation and suppression. For instance, combining regulatory Treg markers (FoxP3) with CD8+ TILs has shown predictive value for immunotherapy responses in various solid tumors [41,42]. Similarly, other combined immunoscores have shown promising results, such as CD3-CD8 combination in colorectal cancer [43] and the combination of TAM markers (CD68 and CD163) alongside T-cell markers in lung cancer [44]. Additionally, the co-expression of multiple immune checkpoints, such as LAG-3, TIM-3, and TIGIT, on exhausted T-cells is being investigated for its predictive value, especially as novel checkpoint inhibitors targeting these pathways enter clinical trials [35,37].

Although these combined approaches offer great promise, there are still many issues to overcome, such as assay standardization among labs, the lack of uniform platforms, the intra-tumor and inter-patient heterogeneity, and the time-dependent fluctuations of the immune markers, before they are clinically validated and subsequently implemented in clinical practice.

## 3. Peripheral Blood Immune Biomarkers

**Figure 2:**
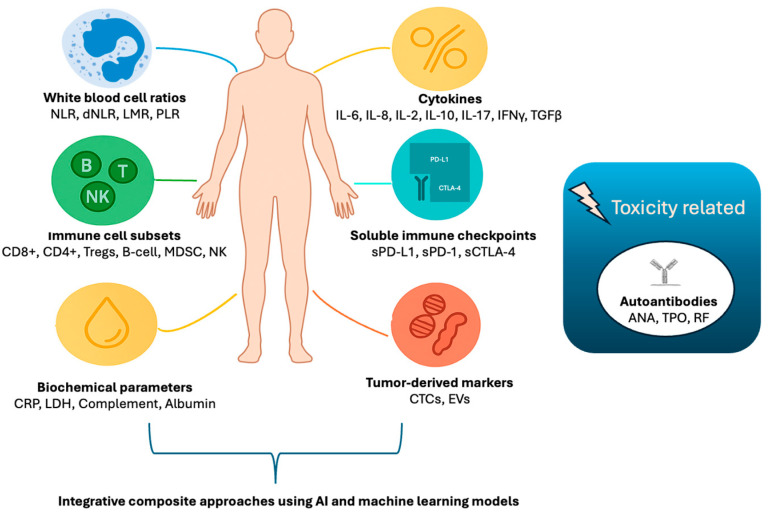
Summary of current and investigatory peripheral blood immune markers in patients treated with immunotherapy. **Abbreviations:** ANA: anti-nuclear antibody; CD: cluster of differentiation; CRP: C-reactive protein; CTC: circulating tumor cells; CTLA-4: cytotoxic T-lymphocyte-associated protein-4; EV: extracellular vesicles; dNLR: derived neutrophil-to-lymphocyte ratio; IFN-γ: interferon-γ; IL: interleukin; LDH: lactate dehydrogenase; LMR: lymphocyte-to-monocyte ratio; MDSC: myeloid-derived suppressor cells; NK: natural killer cells; NLR: neutrophil-to-lymphocyte ratio; PD-1: programmed death receptor-1; PD-L1: programmed death-ligand-1; PLR: platelet-to-lymphocyte ratio; sPD-1/sPD-L1/sCTLA-4: soluble isoforms of the immune checkpoint proteins; RF: rheumatoid factor; TGF-β: tumor growth factor-β; TPO: thyroid peroxidase; Tregs: regulatory T-cells.

Peripheral blood immune biomarkers have emerged as valuable tools for evaluating the immune status of cancer patients and response to ICIs in a minimally invasive manner. Tissue-based markers provide localized and temporal information of the TIME, but peripheral blood markers can offer a dynamic, real-time monitoring into the host’s immune status and its interaction with the tumor. Thus, peripheral markers can be potentially utilized for the prognosis and the monitoring of therapeutic response. This section will review the main categories of peripheral immune biomarkers (Figure 2), ranging from simple cell count ratios to advanced immunophenotyping, highlighting their clinical relevance, emerging evidence, and future prospects. A summary of the currently studied peripheral immune markers is provided in Table 2.

### 3.1. White Blood Cell Ratios and Composite Indices

Ratios derived from routine blood count, such as the neutrophil-to-lymphocyte ratio (NLR), lymphocyte-to-monocyte ratio (LMR), platelet-to-lymphocyte ratio (PLR), and derived NLR (dNLR), are easily accessible and cost-efficient. A combination of different subsets of white blood cells can be utilized to assess the balance between pro-inflammatory and anti-tumors states, which in turn may reflect disease progression and response to therapy. Several studies have shown that an elevated NLR, indicating an increase in neutrophils or decrease in lymphocytes, is associated with worse outcomes in many solid tumors, including lung, BC, and colorectal cancers [45,46]. Similarly, low LMR and high PLR have been linked to unfavorable prognosis [47,48,49]. In the context of immunotherapy, several studies associate these ratios with the prediction of treatment response and immune-related toxicity, suggesting their potential role in patient stratification and treatment monitoring [48,49].

In conjunction to these ratios several other biochemical parameters, reflecting the inflammatory burden, the tumor load, or the nutritional status, have been employed to synthesize composite tools to predict response. The Lung Immune Prognostic Index (LIPI) incorporates dNLR and lactate dehydrogenase (LDH) levels and has demonstrated strong predictive value for survival in NSCLC patients treated with ICIs [50]. The Systemic Immune-Inflammation Index (SII), integrating neutrophil, lymphocyte, and platelet counts, has shown prognostic value across a range of cancers and strong association with response to immunotherapy in lung cancer [51,52]. Additionally, other markers of systemic inflammation, such as C-reactive protein (CRP) or biochemical nutritional markers (e.g., albumin, CRP-to-albumin ratio), have been investigated in composite scores and have provided promising results regarding the prediction of immunotherapy outcomes [53,54]. Moreover, complement components C3 and C4 have also been assessed in multiparametric models, with early findings suggesting that lower levels of C3 or of the ratio C3/C4 may be associated with favorable responses to ICIs [55].

Although these combined indices are promising due to their simplicity and accessibility, further validation and consensus on cutoff values are required. Moreover, these indices are influenced by a variety of factors including infections, medications such as corticosteroids, and comorbidities, which may hamper their specificity.

### 3.2. Circulating Immune Cell Subsets and Immunophenotyping

Although white blood cell ratios have shown promising results in several studies, the extensive profiling of white blood cell subsets and of their functional status may provide a more detailed insight into immune system responses against cancer. Advanced immunophenotyping techniques, such as multiparametric flow cytometry and mass cytometry (CyTOF), allow detailed characterization of these populations, enabling evaluation of their prognostic and predictive significance in patients undergoing immunotherapy with ICIs.

Among the key immune subsets, lymphocytes, particularly CD8+ and CD4+ T-cells, play a central role in anti-tumor immunity. Elevated peripheral CD8+ T-cell counts or high CD4+/CD8+ ratios have been associated with improved outcomes in various cancers treated with ICIs, while in some studies they are associated with increased incidence and severity of irAEs [56,57,58,59]. Moreover, dynamic increases in activated CD8+ T-cells during therapy have been shown to be predictive of anti-cancer outcomes [57]. On the contrary, reduced lymphocyte counts or low CD4+/CD8+ ratios are linked to poor prognosis and diminished immunotherapy benefit [57].

Peripheral cytotoxic and helper T-cells reflect an immunostimulatory population that is associated with better outcomes. On the contrary, the elevated levels of immunosuppressive populations, such as regulatory Tregs and myeloid-derived suppressor cells (MDSCs), correlate with worse outcomes. Elevated peripheral Tregs have been associated with immune escape mechanisms and resistance to ICIs [60]. They have also been associated with therapy success in scenarios where they were monitored as surrogate biomarkers for disease progression [60,61]. Similarly, high peripheral MDSC levels may predict poor survival and reduced response to immunotherapy as these cells inhibit effective T-cell function [62]. Importantly, monitoring reductions in MDSC levels during therapy has been associated with clinical benefit, suggesting their potential as dynamic biomarkers [63].

Natural killer (NK) cells have also been associated with favorable responses to ICIs in early studies. In a recent immunophenotyping study including breast, pancreatic, and HNSCC, a combined immunotype signature including NKs, dendritic cells, and PD-L1+/CD8+ T-cells demonstrated good predictive ability for treatment response (chemotherapy and immunotherapy) and increased NK levels during treatment were proved as a surrogate marker of therapy response [64].

T-cells have been the primary focus of immunophenotyping, there is though emerging evidence supporting the role of B-cells as prognostic and predictive markers. Increased levels of memory B-cells and plasma cells have been associated with better responses and survival in patients with various solid tumors treated with ICIs [65,66,67]. These peripheral B-cell profiles may reflect humoral immunity and mark TLS activity within tumors, which in turn correlates with improved immunotherapy outcomes [33,68]. Moreover, cells participating in the innate immunity may be associated with immunotherapy responses. For instance, helper innate lymphoid cells (hILCs), a type of immune cells contributing to the interplay of adaptive and innate immunity with distinct functions in cytokine regulation mirroring the roles of helper T-cells, have been shown to fluctuate during immunotherapy treatment. Studies in melanoma have demonstrated in-treatment dynamic changes in hILCs and specific subsets of hILCs have been associated with therapy response [69].

### 3.3. Cytokines

Cytokines are soluble proteins that promote communication between the immune cells and regulate the TIME. Their levels in peripheral blood may reflect the status of the immune system, rendering them as potential biomarkers to predict prognosis, therapeutic response, and irAEs in cancer patients undergoing ICI therapy.

Key immune-activating cytokines include interferon-gamma (IFN-γ), tumor necrosis factor-alpha (TNF-α), and interleukin-2 (IL-2). IFN-γ is crucial for enhancing antigen presentation and T-cell cytotoxicity. Elevated baseline or early increases in IFN-γ have been associated with improved responses to ICIs in NSCLC and gastric cancer patients [70]. IL-2 promotes T-cell proliferation and survival, with some studies linking higher circulating IL-2 levels in models also encompassing other pro-inflammatory cytokines (e.g., IL-1, IFN-α) to immunotherapy toxicity [71]. The role of TNF-α is still under investigation. In some studies pre-treatment elevated TNF-α levels were associated with resistance to therapy [72].

In contrast, other cytokines, such as interleukin-6 (IL-6), interleukin-8 (IL-8), transforming growth factor-beta (TGF-β), and interleukin-10 (IL-10) have immunosuppressive effects and contribute to tumor progression. Elevated serum IL-6 and IL-8 levels have been repeatedly associated with poor prognosis and resistance to ICIs [73]. IL-8, in particular, has emerged as a strong predictor of response, with higher baseline levels linked to decreased survival and early disease progression in multiple cancers [73]. It has also been suggested that serum concentrations of TGF-β and IL-10 are associated with therapeutic effects and higher levels of IL-17 can predict irAEs in melanoma patients treated with ipilimumab [71,73].

Strategies for synthesis of different cytokines in composite signatures are gaining attention as potentially improved predictors compared to single cytokine analysis. Multiplex assays and complex panels incorporating both stimulatory and inhibitory molecules have demonstrated better stratification between responders and non-responders [74,75]. These composite profiles may improve early identification of patients at risk for toxicity or treatment failure.

Despite these promising data, cytokine measurement faces several limitations as they present known and unknown pleiotropic effects, their analysis is often difficult due their short half-lives, and their biological diversity makes association with cancer outcomes difficult [76].

### 3.4. Soluble Checkpoint Proteins

More recently, the investigation of several soluble forms of the well-known membrane-bound immune checkpoint proteins, such as PD-1, PD-L1, and CTLA-4 (cytotoxic T-lymphocyte-associated protein-4), as potential blood-based biomarkers has gained attention.

Soluble PD-L1 (sPD-L1) has been on focus since its fluctuating levels have been associated with therapy response in several studies. More specifically, high baseline sPD-L1 concentrations correlate with worse prognosis and response to ICIs, most likely due to the emerging inhibition of T-cell activation in response to rising PD-L1 levels. In particular, high circulating sPD-L1 levels have been associated with shorter overall survival in NSCLC, melanoma, and renal cell carcinoma patients treated with ICIs [77,78,79,80]. Furthermore, emerging data suggest that monitoring sPD-L1 levels during treatment can provide valuable information on therapeutic efficacy and disease progression. For instance, studies on glioma, NSCLC, and melanoma have shown that rising sPD-L1 levels during therapy often precede radiological progression and poor outcomes, whereas stable or decreasing levels are associated with favorable responses [80,81,82].

Similarly, soluble PD-1 and CTLA-4 levels have being explored as predictors of the continuously reforming immune status during treatment, though clinical data remain limited [83,84].

### 3.5. Autoantibodies

Another emerging approach on blood-based biomarkers research is the detection of autoantibodies and their potential role as markers of toxicity and treatment efficacy.

Several studies have demonstrated that pre-existing autoantibodies, including antinuclear antibodies (ANAs), anti-thyroid peroxidase (anti-TPO), and rheumatoid factor (RF), are associated with a higher risk of developing irAEs in patients treated with ICIs [85,86]. The presence of these autoantibodies may identify patients predisposed to autoimmune toxicities during therapy.

Furthermore, ICIs can induce a de novo development of autoantibodies during treatment, often preceding the onset of irAEs [87,88]. This dynamic seroconversion suggests that the monitoring of autoantibody levels may serve as an early surrogate for forthcoming toxicities, allowing timely intervention.

Importantly, some of these studies suggest that the occurrence of irAEs along with the associated seroconversion of autoantibodies may correlate with improved immunotherapy outcomes [67,89].

### 3.6. Emerging Tumor-Derived Circulating Biomarkers

The role of circulating tumor cells (CTCs) and extracellular vesicles (EVs) as potential biomarkers for cancer immunotherapy is also under investigation. These molecules may provide insight into tumor–immune interactions beyond classical immune profiling. CTCs are tumor cells that shed into the bloodstream and can express immune checkpoint molecules such as PD-L1, providing a dynamic measure of tumor status. Elevated PD-L1 expression on CTCs has been correlated with poor prognosis and resistance to ICIs in several cancers, including NSCLC and HNSCC [90,91]. Monitoring CTC phenotypes and their immune-related markers during treatment may help predict response or early relapse, supporting their potential as real-time liquid biopsy tools.

Extracellular vesicles (EVs), including exosomes released by tumor and immune cells, carry proteins, RNA, and immune modulators that may influence the TIME. Tumor-derived EVs expressing PD-L1 have been associated with resistance to ICIs and worse clinical outcomes [92,93]. Furthermore, EV profiling may help with the recognition of other immune checkpoint molecules, cytokines, and miRNAs relevant to immunotherapy efficacy and toxicity [93]. Despite the promising preliminary data, technical challenges in EV isolation and characterization require further studies in the field.

## 4. Composite Approaches and Future Perspectives

### 4.1. Integrated Immune Profiling and Multi-Modal Biomarkers

Recent advances in cancer immunotherapy give a better insight on the complexity of tumor–immune interactions. Subsequently, assessment of single biomarkers seems insufficient to fully explain the complex underlying interplay. As so, integrated immune profiling approaches, combining tissue- and blood-based biomarkers, may provide a more comprehensive picture of the tumors’ immune landscape [40,68,74]. Multimodal strategies aim to improve prognostic and predictive accuracy of the currently used biomarkers using evolving laboratory techniques, such as multiplex immunohistochemistry and digital spatial profiling. These methods allow simultaneous evaluation of multiple immune cell subsets and checkpoint molecules within the TIME [40,75]. In parallel, multimodal cytometric approaches (e.g., high-dimensional flow, mass cytometry) may better characterize the systemic immune phenotypes coupled with multiplex cytokine assays and circulating tumor biomarkers [64,74,94].

Each of these immunophenotyping aspects may complementarily provide more accurate immune signatures for the identification of patients who would benefit from therapy and/or predict timely resistance to treatment [40,64,68,94].

Despite the current limitations (e.g., assays standardization, big data processing), integrated immune profiling represents a critical step toward personalized immunotherapy.

### 4.2. Application of Artificial Intelligence and Machine Learning

Adjunctly to these multimodal strategies, the growing volume and complexity of computational tools render feasible the incorporation of composite biomarkers analysis. Artificial intelligence (AI) and ML techniques have emerged as powerful tools to analyze high-output datasets generated by multiplex tissue imaging, cytometry, proteomics, and genomic profiling [95,96].

AI algorithms can identify complex patterns and predictive signatures not apparent through conventional analyses. For instance, deep learning models applied to digital pathology images have improved quantification of PD-L1 and immune cell subsets, correlating with clinical outcomes more robustly than manual scoring [40,97]. Similarly, ML frameworks integrating peripheral blood immune profiles with soluble biomarkers can predict patient responses and immune-related toxicities [98,99].

As AI tools mature, they revolutionize biomarker research, and they aid precision Oncology by enabling the logistic interpretation of complex data-driven immunoprofiling models.

## 5. Challenges and Future Directions

Despite significant advances in the identification and integration of immune biomarkers from tissue and peripheral blood, several challenges limit their widespread clinical application. A major obstacle is the lack of assay standardization and uniform platform utilization, which leads to variability in measurement and interpretation of the results. This variability complicates comparisons across studies and hampers the establishment of universally accepted thresholds and clinical guidelines.

In addition, heterogeneity and dynamic changes in immune responses pose further difficulties. Immune markers fluctuate over time and may differ substantially between primary tumors and metastatic sites or in peripheral blood. Moreover, fluctuations of immune markers may be seen not only during treatment but also following the circadian rhythm during the day. Multiple immune parameters vary throughout the day and the disruption of circadian homeostasis by immunotherapy may further complicate the in-day and seasonal levels of these markers [100,101]. Defining the optimal timing and frequency for biomarker sampling remains an open question critical for improving prognostic and predictive accuracy.

Integrating multimodal data from tissue and peripheral blood offers a promising path forward but introduces computational and logistical complexity. Handling large datasets demands advanced analytical tools, including AI and ML. Although these technologies enable discovery of novel biomarker signatures and patient stratification models, resolving issues regarding model interpretability and reproducibility is still ahead.

Patient and tumor heterogeneity further complicate biomarker development. Biomarkers must be validated in diverse populations and across tumor types to ensure broad utility. Additionally, clinical implementation faces regulatory, logistical, and cost-effectiveness barriers, which must be addressed to enable routine use.

Next steps for the translation of these multiplex immune parameters into meaningful biomarkers used in clinical routine include their validation both across labs and in the field with large prospective clinical trials. Overcoming these limitations offers the possibility to refine patient selection and improve outcomes in cancer immunotherapy.

## 6. Conclusions

The landscape of immune biomarkers in cancer immunotherapy is continuously growing, moving from established tissue-based markers toward peripheral blood indicators and integrated multimodal approaches. Some tissue markers like PD-L1 and TILs are still fundamental for clinical practice, however peripheral immune biomarkers offer the opportunity for dynamic, minimally invasive monitoring of immune status, which expand our understanding of tumor–immune interactions. The integration of these data, logistically handled by the developing computational tools, may improve patient stratification and guide personalized treatment. Continued research and prospective studies will be essential to translate these advances into routine clinical practice, ultimately improving outcomes for patients receiving immunotherapy.

## Figures and Tables

**Table 1:** Summary of validated and investigatory tissue-based markers of tumor immune microenvironment for immunotherapy in solid tumors.

Biomarker	Type of Marker	Prognostic Value	Predictive Value for Immunotherapy	Clinical Use	Assessment Method	Limitations	Key Tumor Types
**PD-L1** [2,6,9,10,11,12,13,14,15,16,17,18,19,20]	Immune checkpoint protein	Prognostic value varies by context	Validated predictive biomarker for multiple tumor types	Routine clinical use	IHC and digital pathology	Heterogeneous expression, assay variability, and dynamic changes	NSCLC, urothelial, HNSCC, TNBC, gastric, and cervical
**TILs** [3,4,21,22,23,24,25]	Immune cell infiltration	Generally supported by evidence, with tumor-type variability	Emerging predictive marker; standardization ongoing	Recommended (breast cancer) and investigational (others)	IHC and digital pathology	Lack of standardized scoring and spatial heterogeneity	Melanoma, breast cancer, and NSCLC
**Macrophages (M1/M2)** [26,27]	Immune cell infiltration	Potential prognostic relevance; evidence evolving	Preliminary evidence suggests possible predictive role	Experimental	IHC and flow cytometry	Phenotypic plasticity and lack of standardized markers	Various solid tumors
**CAFs** [28]	Stromal cell component	Emerging evidence of association with poor prognosis	Investigational; may influence immunotherapy resistance	Experimental	IHC and multiplex assays	Heterogeneity and lack of standardized markers	NSCLC, skin, and other solid tumors
**Tregs** [29,30,31,32]	Immune cell infiltration	Associated with immune suppression; prognostic impact varies	Investigational predictive role; therapeutic targeting under study	Experimental	IHC and flow cytometry	Heterogeneity and complex roles in tumor immunity	Various solid tumors
**TLS** [33,34]	Organized immune structures	Supported by growing evidence; standardization pending	Emerging predictive marker; clinical validation ongoing	Investigational	IHC and digital pathology	Lack of standardized quantification	Various solid tumors and sarcoma
**LAG-3** [35,36,37]	Immune checkpoint protein	Investigational	Investigational	Experimental	IHC and flow cytometry	Limited assay validation	Various solid tumors
**TIM-3** [35,36,37]	Immune checkpoint protein	Investigational	Investigational	Experimental	IHC and flow cytometry	Limited assay validation	Various solid tumors
**TIGIT** [35,36,37]	Immune checkpoint protein	Investigational	Investigational	Experimental	IHC and flow cytometry	Limited assay validation	Various solid tumors
**Multiplex Immune Markers** [35,36,37,38,39,40,41,42,43,44]	PD-L1/CD8+, FoxP3-CD8+, etc.	Investigational	Predictive, not validated in clinical trails	Experimental	Multiplex techniques (e.g., IF)	Variability in studies and not validated in trials	Various solid tumors

**CAFs:** cancer-associated fibroblasts; **HNSCC:** head and neck squamous cell carcinoma; **IF:** immunofluorescence; **IHC:** immunohistochemistry; **LAG-3:** lymphocyte activation gene-3; **NSCLC:** non-small-cell lung cancer; **PD-L1:** programmed-death ligand 1; **TIGIT:** T-cell immunoreceptor with Ig and ITIM domains; **TILs:** tumor-infiltrating lymphocytes; **TIM-3:** T-cell immunoglobulin and mucin-domain containing-3; **TLS:** tertiary lymphoid structures; **TNBC:** triple-negative breast cancer; **Tregs:** regulatory T-cells.

**Table 2:** Summary of peripheral blood immune biomarkers in cancer immunotherapy.

Biomarker Category	Specific Markers/Indices	Biological Role	Clinical Relevance and Evidence	Limitations
**White Blood Cell Ratios** [45,46,47,48,49]	NLR, LMR, PLR, and dNLR	Reflect systemic inflammation and immune balance	Prognostic and predictive value across multiple tumors; composite scores improve stratification	Affected by infection, medications, comorbidities; lack of standardized cutoffs and timing
**Biochemical Parameters** [50,51,52,53,54,55]	CRP, LDH, complement components (C3, C4), and albumin	Markers of systemic inflammation, nutritional status, and immune activation	Included in composite scores; complement proteins emerging as immune modulators; albumin reflects nutritional/immune status	Influenced by non-cancer factors (infection, nutrition); need for further validation
**Immune Cell Subsets** [56,57,58,59,60,61,62,63,64,65,66,67,68,69]	CD8+ T-cells, CD4+ T-cells, Tregs, MDSCs, NK cells, B-cells, and hILCs	Effector, regulatory, suppressive, and innate immunity	Predictive and prognostic significance; dynamic changes during therapy correlate with response and toxicity	Complex analysis; need for assay standardization and prospective validation
**Cytokines** [70,71,72,73,74]	IFN-γ, IL-2, IL-6, IL-8, TNF-α, TGF-β, IL-10, and IL-17	Immune activation or suppression through signaling	Baseline and dynamic levels predict response, survival, and irAEs; composite cytokine signatures promising	Biological variability; assay standardization needed; pleiotropic effects
**Soluble Checkpoint Proteins** [75,76,77,78,79,80,81,82]	sPD-L1, sPD-1, and sCTLA-4	Modulate immune checkpoint pathways systemically	Elevated sPD-L1 linked to poor prognosis and resistance; dynamic changes correlate with therapy response	Assay variability; unclear biological functions of soluble vs. membrane forms
**Autoantibodies** [83,84,85,86,87]	ANA, anti-TPO, rheumatoid factor, and others	Reflect autoimmunity and immune activation	Associated with immune-related adverse events; possible link to treatment efficacy	Variability in assays; heterogeneity of targets; clinical utility still investigational
**Tumor-Derived Circulating Biomarkers** [88,89,90,91]	CTCs and EVs	Reflect tumor burden, immune evasion via checkpoint expression	CTC PD-L1 expression and PD-L1+ EVs correlate with resistance and prognosis; promising for monitoring	Technical challenges in isolation, characterization, and standardization
**Integrative Composite Approaches** [40,64,68,92,93,94]	Multi-modal biomarker panels combining tissue, peripheral blood, soluble factors, and tumor-derived markers	Capture complex immune landscapes and tumor heterogeneity	Composite immunoscores improve prediction of immunotherapy response; integration of AI/ML enhances biomarker discovery	Data harmonization, standardization, and clinical validation remain significant

**ANA:** anti-nuclear antibody; **CD:** cluster of differentiation; **CRP:** C-reactive protein; **CTC:** circulating tumor cells; **CTLA-4:** cytotoxic T-lymphocyte-associated protein-4; **EV:** extracellular vesicles; **dNLR:** derived neutrophil-to-lymphocyte ratio; **hILCs:** helper innate lymphoid cells; **IFN-γ:** interferon-γ; **IL:** interleukin; **LDH:** lactate dehydrogenase; **LMR:** lymphocyte-to-monocyte ratio; **MDSC:** myeloid-derived suppressor cells; **NK:** natural killer cells; **NLR:** neutrophil-to-lymphocyte ratio; **PD-1:** programmed death receptor-1; **PD-L1:** programmed death-ligand-1; **PLR:** platelet-to-lymphocyte ratio; **sPD-1/sPD-L1/sCTLA-4:** soluble isoforms of the immune checkpoint proteins; **TGF-β:** tumor growth factor-β; **TNF-α:** tumor necrosis factor-α; **TPO:** thyroid peroxidase; **Tregs:** regulatory T-cells.

## Data Availability

This study is a narrative review and does not contain original experimental data. All information synthesized and referenced is sourced from publicly available literature as cited throughout the manuscript.

## References

[1] Jamalinia M., Weiskirchen R. (2025). Advances in personalized medicine: Translating genomic insights into targeted therapies for cancer treatment. Ann. Transl. Med..

[2] Patel S.P., Kurzrock R. (2015). PD-L1 Expression as a Predictive Biomarker in Cancer Immunotherapy. Mol. Cancer Ther..

[3] Azimi F., Scolyer R.A., Rumcheva P., Moncrieff M., Murali R., McCarthy S.W., Saw R.P., Thompson J.F. (2012). Tumor-infiltrating lymphocyte grade is an independent predictor of sentinel lymph node status and survival in patients with cutaneous melanoma. J. Clin. Oncol..

[4] Denkert C., Loibl S., Noske A., Roller M., Muller B.M., Komor M., Budczies J., Darb-Esfahani S., Kronenwett R., Hanusch C. (2010). Tumor-associated lymphocytes as an independent predictor of response to neoadjuvant chemotherapy in breast cancer. J. Clin. Oncol..

[5] Yamaguchi H., Hsu J.M., Sun L., Wang S.C., Hung M.C. (2024). Advances and prospects of biomarkers for immune checkpoint inhibitors. Cell Rep. Med..

[6] Ilie M., Long-Mira E., Bence C., Butori C., Lassalle S., Bouhlel L., Fazzalari L., Zahaf K., Lalvee S., Washetine K. (2016). Comparative study of the PD-L1 status between surgically resected specimens and matched biopsies of NSCLC patients reveal major discordances: A potential issue for anti-PD-L1 therapeutic strategies. Ann. Oncol..

[7] Goswami M., Toney N.J., Pitts S.C., Celades C., Schlom J., Donahue R.N. (2024). Peripheral immune biomarkers for immune checkpoint inhibition of solid tumours. Clin. Transl. Med..

[8] Nixon A.B., Schalper K.A., Jacobs I., Potluri S., Wang I.M., Fleener C. (2019). Peripheral immune-based biomarkers in cancer immunotherapy: Can we realize their predictive potential?. J. Immunother. Cancer.

[9] Reck M., Rodriguez-Abreu D., Robinson A.G., Hui R., Csoszi T., Fulop A., Gottfried M., Peled N., Tafreshi A., Cuffe S. (2016). Pembrolizumab versus Chemotherapy for PD-L1-Positive Non-Small-Cell Lung Cancer. N. Engl. J. Med..

[10] Rosenberg J.E., Hoffman-Censits J., Powles T., van der Heijden M.S., Balar A.V., Necchi A., Dawson N., O’Donnell P.H., Balmanoukian A., Loriot Y. (2016). Atezolizumab in patients with locally advanced and metastatic urothelial carcinoma who have progressed following treatment with platinum-based chemotherapy: A single-arm, multicentre, phase 2 trial. Lancet.

[11] Burtness B., Harrington K.J., Greil R., Soulieres D., Tahara M., de Castro G., Psyrri A., Baste N., Neupane P., Bratland A. (2019). Pembrolizumab alone or with chemotherapy versus cetuximab with chemotherapy for recurrent or metastatic squamous cell carcinoma of the head and neck (KEYNOTE-048): A randomised, open-label, phase 3 study. Lancet.

[12] Cortes J., Cescon D.W., Rugo H.S., Nowecki Z., Im S.A., Yusof M.M., Gallardo C., Lipatov O., Barrios C.H., Holgado E. (2020). Pembrolizumab plus chemotherapy versus placebo plus chemotherapy for previously untreated locally recurrent inoperable or metastatic triple-negative breast cancer (KEYNOTE-355): A randomised, placebo-controlled, double-blind, phase 3 clinical trial. Lancet.

[13] Shitara K., Van Cutsem E., Bang Y.J., Fuchs C., Wyrwicz L., Lee K.W., Kudaba I., Garrido M., Chung H.C., Lee J. (2020). Efficacy and Safety of Pembrolizumab or Pembrolizumab Plus Chemotherapy vs Chemotherapy Alone for Patients With First-line, Advanced Gastric Cancer: The KEYNOTE-062 Phase 3 Randomized Clinical Trial. JAMA Oncol..

[14] Chung H.C., Ros W., Delord J.P., Perets R., Italiano A., Shapira-Frommer R., Manzuk L., Piha-Paul S.A., Xu L., Zeigenfuss S. (2019). Efficacy and Safety of Pembrolizumab in Previously Treated Advanced Cervical Cancer: Results From the Phase II KEYNOTE-158 Study. J. Clin. Oncol..

[15] Wu P., Wu D., Li L., Chai Y., Huang J. (2015). PD-L1 and Survival in Solid Tumors: A Meta-Analysis. PLoS ONE.

[16] Hendry S., Byrne D.J., Wright G.M., Young R.J., Sturrock S., Cooper W.A., Fox S.B. (2018). Comparison of Four PD-L1 Immunohistochemical Assays in Lung Cancer. J. Thorac. Oncol..

[17] Choe E.A., Cha Y.J., Kim J.H., Pyo K.H., Hong M.H., Park S.Y., Shim H.S., Jung I., Lee C.Y., Cho B.C. (2019). Dynamic changes in PD-L1 expression and CD8^+^ T cell infiltration in non-small cell lung cancer following chemoradiation therapy. Lung Cancer.

[18] van Eekelen L., Spronck J., Looijen-Salamon M., Vos S., Munari E., Girolami I., Eccher A., Acs B., Boyaci C., de Souza G.S. (2024). Comparing deep learning and pathologist quantification of cell-level PD-L1 expression in non-small cell lung cancer whole-slide images. Sci. Rep..

[19] Baxi V., Lee G., Duan C., Pandya D., Cohen D.N., Edwards R., Chang H., Li J., Elliott H., Pokkalla H. (2022). Association of artificial intelligence-powered and manual quantification of programmed death-ligand 1 (PD-L1) expression with outcomes in patients treated with nivolumab +/− ipilimumab. Mod. Pathol..

[20] McGenity C., Clarke E.L., Jennings C., Matthews G., Cartlidge C., Freduah-Agyemang H., Stocken D.D., Treanor D. (2024). Artificial intelligence in digital pathology: A systematic review and meta-analysis of diagnostic test accuracy. NPJ Digit. Med..

[21] Tumeh P.C., Harview C.L., Yearley J.H., Shintaku I.P., Taylor E.J., Robert L., Chmielowski B., Spasic M., Henry G., Ciobanu V. (2014). PD-1 blockade induces responses by inhibiting adaptive immune resistance. Nature.

[22] Yan Q., Li S., He L., Chen N. (2024). Prognostic implications of tumor-infiltrating lymphocytes in non-small cell lung cancer: A systematic review and meta-analysis. Front. Immunol..

[23] Jiang P., Gu S., Pan D., Fu J., Sahu A., Hu X., Li Z., Traugh N., Bu X., Li B. (2018). Signatures of T cell dysfunction and exclusion predict cancer immunotherapy response. Nat. Med..

[24] Hendry S., Salgado R., Gevaert T., Russell P.A., John T., Thapa B., Christie M., van de Vijver K., Estrada M.V., Gonzalez-Ericsson P.I. (2017). Assessing Tumor-Infiltrating Lymphocytes in Solid Tumors: A Practical Review for Pathologists and Proposal for a Standardized Method from the International Immuno-Oncology Biomarkers Working Group: Part 2: TILs in Melanoma, Gastrointestinal Tract Carcinomas, Non-Small Cell Lung Carcinoma and Mesothelioma, Endometrial and Ovarian Carcinomas, Squamous Cell Carcinoma of the Head and Neck, Genitourinary Carcinomas, and Primary Brain Tumors. Adv. Anat. Pathol..

[25] Salgado R., Denkert C., Demaria S., Sirtaine N., Klauschen F., Pruneri G., Wienert S., Van den Eynden G., Baehner F.L., Penault-Llorca F. (2015). The evaluation of tumor-infiltrating lymphocytes (TILs) in breast cancer: Recommendations by an International TILs Working Group 2014. Ann. Oncol..

[26] Lin Y., Xu J., Lan H. (2019). Tumor-associated macrophages in tumor metastasis: Biological roles and clinical therapeutic applications. J. Hematol. Oncol..

[27] Wei C., Ma Y., Wang M., Wang S., Yu W., Dong S., Deng W., Bie L., Zhang C., Shen W. (2024). Tumor-associated macrophage clusters linked to immunotherapy in a pan-cancer census. NPJ Precis. Oncol..

[28] Peyraud F., Guegan J.P., Rey C., Lara O., Odin O., Del Castillo M., Vanhersecke L., Coindre J.M., Clot E., Brunet M. (2025). Spatially resolved transcriptomics reveal the determinants of primary resistance to immunotherapy in NSCLC with mature tertiary lymphoid structures. Cell Rep. Med..

[29] Bates G.J., Fox S.B., Han C., Leek R.D., Garcia J.F., Harris A.L., Banham A.H. (2006). Quantification of regulatory T cells enables the identification of high-risk breast cancer patients and those at risk of late relapse. J. Clin. Oncol..

[30] Plitas G., Konopacki C., Wu K., Bos P.D., Morrow M., Putintseva E.V., Chudakov D.M., Rudensky A.Y. (2016). Regulatory T Cells Exhibit Distinct Features in Human Breast Cancer. Immunity.

[31] Sharma P., Hu-Lieskovan S., Wargo J.A., Ribas A. (2017). Primary, Adaptive, and Acquired Resistance to Cancer Immunotherapy. Cell.

[32] Qin D., Zhang Y., Shu P., Lei Y., Li X., Wang Y. (2024). Targeting tumor-infiltrating tregs for improved antitumor responses. Front. Immunol..

[33] Sautes-Fridman C., Petitprez F., Calderaro J., Fridman W.H. (2019). Tertiary lymphoid structures in the era of cancer immunotherapy. Nat. Rev. Cancer.

[34] Italiano A., Bessede A., Pulido M., Bompas E., Piperno-Neumann S., Chevreau C., Penel N., Bertucci F., Toulmonde M., Bellera C. (2022). Pembrolizumab in soft-tissue sarcomas with tertiary lymphoid structures: A phase 2 PEMBROSARC trial cohort. Nat. Med..

[35] Andrews L.P., Marciscano A.E., Drake C.G., Vignali D.A. (2017). LAG3 (CD223) as a cancer immunotherapy target. Immunol. Rev..

[36] Anderson A.C. (2012). Tim-3, a negative regulator of anti-tumor immunity. Curr. Opin. Immunol..

[37] Chauvin J.M., Zarour H.M. (2020). TIGIT in cancer immunotherapy. J. Immunother. Cancer.

[38] Taube J.M., Anders R.A., Young G.D., Xu H., Sharma R., McMiller T.L., Chen S., Klein A.P., Pardoll D.M., Topalian S.L. (2012). Colocalization of inflammatory response with B7-h1 expression in human melanocytic lesions supports an adaptive resistance mechanism of immune escape. Sci. Transl. Med..

[39] Herbst R.S., Soria J.C., Kowanetz M., Fine G.D., Hamid O., Gordon M.S., Sosman J.A., McDermott D.F., Powderly J.D., Gettinger S.N. (2014). Predictive correlates of response to the anti-PD-L1 antibody MPDL3280A in cancer patients. Nature.

[40] Thommen D.S., Koelzer V.H., Herzig P., Roller A., Trefny M., Dimeloe S., Kiialainen A., Hanhart J., Schill C., Hess C. (2018). A transcriptionally and functionally distinct PD-1^+^ CD8^+^ T cell pool with predictive potential in non-small-cell lung cancer treated with PD-1 blockade. Nat. Med..

[41] Yamagami W., Susumu N., Tanaka H., Hirasawa A., Banno K., Suzuki N., Tsuda H., Tsukazaki K., Aoki D. (2011). Immunofluorescence-detected infiltration of CD4^+^FOXP3^+^ regulatory T cells is relevant to the prognosis of patients with endometrial cancer. Int. J. Gynecol. Cancer.

[42] Furgiuele S., Descamps G., Lechien J.R., Dequanter D., Journe F., Saussez S. (2022). Immunoscore Combining CD8, FoxP3, and CD68-Positive Cells Density and Distribution Predicts the Prognosis of Head and Neck Cancer Patients. Cells.

[43] Angell H.K., Bruni D., Barrett J.C., Herbst R., Galon J. (2020). The Immunoscore: Colon Cancer and Beyond. Clin. Cancer Res..

[44] Wu X.R., Peng H.X., He M., Zhong R., Liu J., Wen Y.K., Li C.C., Li J.F., Xiong S., Yu T. (2022). Macrophages-based immune-related risk score model for relapse prediction in stage I-III non-small cell lung cancer assessed by multiplex immunofluorescence. Transl. Lung Cancer Res..

[45] Templeton A.J., McNamara M.G., Seruga B., Vera-Badillo F.E., Aneja P., Ocana A., Leibowitz-Amit R., Sonpavde G., Knox J.J., Tran B. (2014). Prognostic role of neutrophil-to-lymphocyte ratio in solid tumors: A systematic review and meta-analysis. J. Natl. Cancer Inst..

[46] Su J., Li Y., Tan S., Cheng T., Luo Y., Zhang L. (2025). Pretreatment neutrophil-to-lymphocyte ratio is associated with immunotherapy efficacy in patients with advanced cancer: A systematic review and meta-analysis. Sci. Rep..

[47] Diem S., Schmid S., Krapf M., Flatz L., Born D., Jochum W., Templeton A.J., Fruh M. (2017). Neutrophil-to-Lymphocyte ratio (NLR) and Platelet-to-Lymphocyte ratio (PLR) as prognostic markers in patients with non-small cell lung cancer (NSCLC) treated with nivolumab. Lung Cancer.

[48] Zhang N., Jiang J., Tang S., Sun G. (2020). Predictive value of neutrophil-lymphocyte ratio and platelet-lymphocyte ratio in non-small cell lung cancer patients treated with immune checkpoint inhibitors: A meta-analysis. Int. Immunopharmacol..

[49] Wan L., Wu C., Luo S., Xie X. (2022). Prognostic Value of Lymphocyte-to-Monocyte Ratio (LMR) in Cancer Patients Undergoing Immune Checkpoint Inhibitors. Dis. Markers.

[50] Mezquita L., Auclin E., Ferrara R., Charrier M., Remon J., Planchard D., Ponce S., Ares L.P., Leroy L., Audigier-Valette C. (2018). Association of the Lung Immune Prognostic Index With Immune Checkpoint Inhibitor Outcomes in Patients With Advanced Non-Small Cell Lung Cancer. JAMA Oncol..

[51] Yang R., Chang Q., Meng X., Gao N., Wang W. (2018). Prognostic value of Systemic immune-inflammation index in cancer: A meta-analysis. J. Cancer.

[52] Zhang Y., Chen Y., Guo C., Li S., Huang C. (2025). Systemic immune-inflammation index as a predictor of survival in non-small cell lung cancer patients undergoing immune checkpoint inhibition: A systematic review and meta-analysis. Crit. Rev. Oncol. Hematol..

[53] Dai M., Wu W. (2023). Prognostic role of C-reactive protein to albumin ratio in cancer patients treated with immune checkpoint inhibitors: A meta-analysis. Front. Oncol..

[54] Tong W., Xu H., Tang J., Zhao N., Zhou D., Chen C., Cao D. (2024). Inflammatory factors are associated with prognosis of non-small cell lung cancer patients receiving immunotherapy: A meta-analysis. Sci. Rep..

[55] Krizova L., Benesova I., Zemanova P., Spacek J., Strizova Z., Humlova Z., Mikulova V., Petruzelka L., Vocka M. (2024). Immunophenotyping of peripheral blood in NSCLC patients discriminates responders to immune checkpoint inhibitors. J. Cancer Res. Clin. Oncol..

[56] Miao K., Zhang X., Wang H., Si X., Ni J., Zhong W., Zhao J., Xu Y., Chen M., Pan R. (2022). Peripheral Blood Lymphocyte Subsets Predict the Efficacy of Immune Checkpoint Inhibitors in Non-Small Cell Lung Cancer. Front. Immunol..

[57] Lao J., Xu H., Liang Z., Luo C., Shu L., Xie Y., Wu Y., Hao Y., Yuan Y. (2023). Peripheral changes in T cells predict efficacy of anti-PD-1 immunotherapy in non-small cell lung cancer. Immunobiology.

[58] Xu S., Zhu Q., Wu L., Wang Y., Wang J., Zhu L., Zheng S., Hang J. (2023). Association of the CD4^+^/CD8^+^ ratio with response to PD-1 inhibitor-based combination therapy and dermatological toxicities in patients with advanced gastric and esophageal cancer. Int. Immunopharmacol..

[59] Li P., Qin P., Fu X., Zhang G., Yan X., Zhang M., Zhang X., Yang J., Wang H., Ma Z. (2021). Associations between peripheral blood lymphocyte subsets and clinical outcomes in patients with lung cancer treated with immune checkpoint inhibitor. Ann. Palliat. Med..

[60] Huang S.W., Jiang W., Xu S., Zhang Y., Du J., Wang Y.Q., Yang K.Y., Zhang N., Liu F., Zou G.R. (2024). Systemic longitudinal immune profiling identifies proliferating Treg cells as predictors of immunotherapy benefit: Biomarker analysis from the phase 3 CONTINUUM and DIPPER trials. Signal Transduct. Target. Ther..

[61] Kang D.H., Chung C., Sun P., Lee D.H., Lee S.I., Park D., Koh J.S., Kim Y., Yi H.S., Lee J.E. (2022). Circulating regulatory T cells predict efficacy and atypical responses in lung cancer patients treated with PD-1/PD-L1 inhibitors. Cancer Immunol. Immunother..

[62] Moller M., Orth V., Umansky V., Hetjens S., Braun V., Reissfelder C., Hardt J., Seyfried S. (2024). Myeloid-derived suppressor cells in peripheral blood as predictive biomarkers in patients with solid tumors undergoing immune checkpoint therapy: Systematic review and meta-analysis. Front. Immunol..

[63] Gaissler A., Bochem J., Spreuer J., Ottmann S., Martens A., Amaral T., Wagner N.B., Claassen M., Meier F., Terheyden P. (2023). Early decrease of blood myeloid-derived suppressor cells during checkpoint inhibition is a favorable biomarker in metastatic melanoma. J. Immunother. Cancer.

[64] Dyikanov D., Zaitsev A., Vasileva T., Wang I., Sokolov A.A., Bolshakov E.S., Frank A., Turova P., Golubeva O., Gantseva A. (2024). Comprehensive peripheral blood immunoprofiling reveals five immunotypes with immunotherapy response characteristics in patients with cancer. Cancer Cell.

[65] Yuan S., Liu Y., Till B., Song Y., Wang Z. (2020). Pretreatment Peripheral B Cells Are Associated With Tumor Response to Anti-PD-1-Based Immunotherapy. Front. Immunol..

[66] Barth D.A., Stanzer S., Spiegelberg J.A., Bauernhofer T., Absenger G., Szkandera J., Gerger A., Smolle M.A., Hutterer G.C., Ahyai S.A. (2022). Patterns of Peripheral Blood B-Cell Subtypes Are Associated With Treatment Response in Patients Treated With Immune Checkpoint Inhibitors: A Prospective Longitudinal Pan-Cancer Study. Front. Immunol..

[67] Willsmore Z.N., Booth L., Patel A., Di Meo A., Prassas I., Chauhan J., Wu Y., Fitzpartick A., Stoker K., Kapiris M. (2025). Circulating immunoregulatory B cell and autoreactive antibody profiles predict lack of toxicity to anti-PD-1 checkpoint inhibitor treatment in advanced melanoma. J. Immunother. Cancer.

[68] Helmink B.A., Reddy S.M., Gao J., Zhang S., Basar R., Thakur R., Yizhak K., Sade-Feldman M., Blando J., Han G. (2020). B cells and tertiary lymphoid structures promote immunotherapy response. Nature.

[69] Cristiani C.M., Capone M., Garofalo C., Madonna G., Mallardo D., Tuffanelli M., Vanella V., Greco M., Foti D.P., Viglietto G. (2022). Altered frequencies and functions of innate lymphoid cells in melanoma patients are modulated by immune checkpoints inhibitors. Front. Immunol..

[70] Zhao Q., Bi Y., Sun H., Xiao M. (2021). Serum IL-5 and IFN-gamma Are Novel Predictive Biomarkers for Anti-PD-1 Treatment in NSCLC and GC Patients. Dis. Markers.

[71] Lim S.Y., Lee J.H., Gide T.N., Menzies A.M., Guminski A., Carlino M.S., Breen E.J., Yang J.Y.H., Ghazanfar S., Kefford R.F. (2019). Circulating Cytokines Predict Immune-Related Toxicity in Melanoma Patients Receiving Anti-PD-1-Based Immunotherapy. Clin. Cancer Res..

[72] Hardy-Werbin M., Rocha P., Arpi O., Taus A., Nonell L., Duran X., Villanueva X., Joseph-Pietras D., Nolan L., Danson S. (2019). Serum cytokine levels as predictive biomarkers of benefit from ipilimumab in small cell lung cancer. Oncoimmunology.

[73] Mao X.C., Yang C.C., Yang Y.F., Yan L.J., Ding Z.N., Liu H., Yan Y.C., Dong Z.R., Wang D.X., Li T. (2022). Peripheral cytokine levels as novel predictors of survival in cancer patients treated with immune checkpoint inhibitors: A systematic review and meta-analysis. Front. Immunol..

[74] Ji S., Chen H., Yang K., Zhang G., Mao B., Hu Y., Zhang H., Xu J. (2020). Peripheral cytokine levels as predictive biomarkers of benefit from immune checkpoint inhibitors in cancer therapy. Biomed. Pharmacother..

[75] Frigola X., Inman B.A., Lohse C.M., Krco C.J., Cheville J.C., Thompson R.H., Leibovich B., Blute M.L., Dong H., Kwon E.D. (2011). Identification of a soluble form of B7-H1 that retains immunosuppressive activity and is associated with aggressive renal cell carcinoma. Clin. Cancer Res..

[76] Murakami S., Shibaki R., Matsumoto Y., Yoshida T., Goto Y., Kanda S., Horinouchi H., Fujiwara Y., Yamamoto N., Ohe Y. (2020). Association between serum level soluble programmed cell death ligand 1 and prognosis in patients with non-small cell lung cancer treated with anti-PD-1 antibody. Thorac. Cancer.

[77] Brun S.S., Hansen T.F., Wen S.W.C., Nyhus C.H., Bertelsen L., Jakobsen A., Hansen T.S., Nederby L. (2024). Soluble programmed death ligand 1 as prognostic biomarker in non-small cell lung cancer patients receiving nivolumab, pembrolizumab or atezolizumab therapy. Sci. Rep..

[78] Oya K., Nakamura Y., Shen L.T., Ishizuki S., Matsusaka S., Fujisawa Y. (2024). Soluble PD-L1 predicts tumor response and immune-related adverse events in patients with advanced melanoma treated with anti-PD-1 antibodies. J. Dermatol..

[79] Liu S., Zhu Y., Zhang C., Meng X., Sun B., Zhang G., Fan Y., Kang X. (2020). The Clinical Significance of Soluble Programmed Cell Death-Ligand 1 (sPD-L1) in Patients With Gliomas. Front. Oncol..

[80] Himuro H., Nakahara Y., Igarashi Y., Kouro T., Higashijima N., Matsuo N., Murakami S., Wei F., Horaguchi S., Tsuji K. (2023). Clinical roles of soluble PD-1 and PD-L1 in plasma of NSCLC patients treated with immune checkpoint inhibitors. Cancer Immunol. Immunother..

[81] Ohkuma R., Ieguchi K., Watanabe M., Takayanagi D., Goshima T., Onoue R., Hamada K., Kubota Y., Horiike A., Ishiguro T. (2021). Increased Plasma Soluble PD-1 Concentration Correlates with Disease Progression in Patients with Cancer Treated with Anti-PD-1 Antibodies. Biomedicines.

[82] Pistillo M.P., Fontana V., Morabito A., Dozin B., Laurent S., Carosio R., Banelli B., Ferrero F., Spano L., Tanda E. (2019). Soluble CTLA-4 as a favorable predictive biomarker in metastatic melanoma patients treated with ipilimumab: An Italian melanoma intergroup study. Cancer Immunol. Immunother..

[83] Daban A., Gonnin C., Phan L., Saldmann A., Granier C., Lillo-Lelouet A., Le Beller C., Pouchot J., Weiss L., Tartour E. (2023). Preexisting autoantibodies as predictor of immune related adverse events (irAEs) for advanced solid tumors treated with immune checkpoint inhibitors (ICIs). Oncoimmunology.

[84] Genta S., Lajkosz K., Yee N.R., Spiliopoulou P., Heirali A., Hansen A.R., Siu L.L., Saibil S., Stayner L.A., Yanekina M. (2023). Autoimmune PaneLs as PrEdictors of Toxicity in Patients TReated with Immune Checkpoint InhibiTors (ALERT). J. Exp. Clin. Cancer Res..

[85] de Moel E.C., Rozeman E.A., Kapiteijn E.H., Verdegaal E.M.E., Grummels A., Bakker J.A., Huizinga T.W.J., Haanen J.B., Toes R.E.M., van der Woude D. (2019). Autoantibody Development under Treatment with Immune-Checkpoint Inhibitors. Cancer Immunol. Res..

[86] Giannicola R., D’Arrigo G., Botta C., Agostino R., Del Medico P., Falzea A.C., Barbieri V., Staropoli N., Del Giudice T., Pastina P. (2019). Early blood rise in auto-antibodies to nuclear and smooth muscle antigens is predictive of prolonged survival and autoimmunity in metastatic-non-small cell lung cancer patients treated with PD-1 immune-check point blockade by nivolumab. Mol. Clin. Oncol..

[87] Osorio J.C., Ni A., Chaft J.E., Pollina R., Kasler M.K., Stephens D., Rodriguez C., Cambridge L., Rizvi H., Wolchok J.D. (2017). Antibody-mediated thyroid dysfunction during T-cell checkpoint blockade in patients with non-small-cell lung cancer. Ann. Oncol..

[88] Kallergi G., Vetsika E.K., Aggouraki D., Lagoudaki E., Koutsopoulos A., Koinis F., Katsarlinos P., Trypaki M., Messaritakis I., Stournaras C. (2018). Evaluation of PD-L1/PD-1 on circulating tumor cells in patients with advanced non-small cell lung cancer. Ther. Adv. Med. Oncol..

[89] Strati A., Koutsodontis G., Papaxoinis G., Angelidis I., Zavridou M., Economopoulou P., Kotsantis I., Avgeris M., Mazel M., Perisanidis C. (2017). Prognostic significance of PD-L1 expression on circulating tumor cells in patients with head and neck squamous cell carcinoma. Ann. Oncol..

[90] Chen G., Huang A.C., Zhang W., Zhang G., Wu M., Xu W., Yu Z., Yang J., Wang B., Sun H. (2018). Exosomal PD-L1 contributes to immunosuppression and is associated with anti-PD-1 response. Nature.

[91] Poggio M., Hu T., Pai C.C., Chu B., Belair C.D., Chang A., Montabana E., Lang U.E., Fu Q., Fong L. (2019). Suppression of Exosomal PD-L1 Induces Systemic Anti-tumor Immunity and Memory. Cell.

[92] Harel M., Lahav C., Jacob E., Dahan N., Sela I., Elon Y., Raveh Shoval S., Yahalom G., Kamer I., Zer A. (2022). Longitudinal plasma proteomic profiling of patients with non-small cell lung cancer undergoing immune checkpoint blockade. J. Immunother. Cancer.

[93] Hu Y., Li S., Xiao H., Xiong Y., Lu X., Yang X., Luo W., Luo J., Zhang S., Cheng Y. (2023). Distinct circulating cytokine/chemokine profiles correlate with clinical benefit of immune checkpoint inhibitor monotherapy and combination therapy in advanced non-small cell lung cancer. Cancer Med..

[94] Petitprez F., de Reynies A., Keung E.Z., Chen T.W., Sun C.M., Calderaro J., Jeng Y.M., Hsiao L.P., Lacroix L., Bougouin A. (2020). B cells are associated with survival and immunotherapy response in sarcoma. Nature.

[95] Hu J., Li X., Coleman K., Schroeder A., Ma N., Irwin D.J., Lee E.B., Shinohara R.T., Li M. (2021). SpaGCN: Integrating gene expression, spatial location and histology to identify spatial domains and spatially variable genes by graph convolutional network. Nat. Methods.

[96] Ren H., Walker B.L., Cang Z., Nie Q. (2022). Identifying multicellular spatiotemporal organization of cells with SpaceFlow. Nat. Commun..

[97] Saltz J., Gupta R., Hou L., Kurc T., Singh P., Nguyen V., Samaras D., Shroyer K.R., Zhao T., Batiste R. (2018). Spatial Organization and Molecular Correlation of Tumor-Infiltrating Lymphocytes Using Deep Learning on Pathology Images. Cell Rep..

[98] Zhang H., Wu W., Wang M., Zhang J., Guo C., Han G., Wang L. (2025). Integrated peripheral blood multi-omics profiling identifies immune signatures predictive of neoadjuvant PD-1 blockade efficacy in head and neck squamous cell carcinoma. J. Transl. Med..

[99] Wei C., Wang M., Gao Q., Yuan S., Deng W., Bie L., Ma Y., Zhang C., Li S., Luo S. (2023). Dynamic peripheral blood immune cell markers for predicting the response of patients with metastatic cancer to immune checkpoint inhibitors. Cancer Immunol. Immunother..

[100] Wang C., Lutes L., Barnoud C., Scheiermann C. (2022). The circadian immune system. Sci. Immunol..

[101] Scheiermann C., Gibbs J., Ince L., Ludon A. (2018). Clocking in to immunity. Nat. Rev. Immunol..

